# Insulin Restores an Altered Corneal Epithelium Circadian Rhythm in Mice with Streptozotocin-induced Type 1 Diabetes

**DOI:** 10.1038/srep32871

**Published:** 2016-09-09

**Authors:** Fang Song, Yunxia Xue, Dong Dong, Jun Liu, Ting Fu, Chengju Xiao, Hanqing Wang, Cuipei Lin, Peng Liu, Jiajun Zhong, Yabing Yang, Zhaorui Wang, Hongwei Pan, Jiansu Chen, Yangqiu Li, Dongqing Cai, Zhijie Li

**Affiliations:** 1 Key Laboratory for Regenerative Medicine of Ministry of Education, Jinan University, Guangzhou 510632, P.R. China; 2 International Ocular Surface Research Centre and Institute of Ophthalmology, Jinan University Medical School, Guangzhou 510632, P.R. China; 3 Department of Medical Images, The Third People’s Hospital, Puyang, China; 4 Section of Leukocyte Biology, Department of Pediatrics, Children’s Nutrition Research Center, Baylor College of Medicine, Houston, Texas, USA.

## Abstract

The mechanisms of corneal epithelial lesions and delayed wound repair, as well as their association with diabetes mellitus, are critical issues for clinical ophthalmologists. To test whether the diabetic condition alters the circadian rhythm in a mouse cornea and whether insulin can synchronise the corneal clock, we studied the effects of streptozotocin-induced diabetes on the mitosis of epithelial cells, the recruitment of leukocytes to the cornea, and the expression of main core clock genes (*Clock*, *Bmal1*, *Per2*, *Cry1*, and *Rev-erbα*) in the corneal epithelium. We also assessed the possible effect of insulin on these modifications. Diabetes downregulated *Clock*, *Bmal1*, and *Per2* expression, upregulated *Cry1* and *Rev-erbα* expression, reduced corneal epithelial mitosis, and increased leukocyte (neutrophils and γδ T-cells) recruitment to the cornea. Early treatments with insulin partially restored the altered rhythmicity in the diabetic cornea. In conclusion, insulin-dependent diabetes altered the normal rhythmicity of the cornea, and insulin administration had a beneficial effect on restoring normal rhythmicity in the diabetic cornea.

Diabetes mellitus is a metabolic disorder that involves high long-term levels of blood glucose. This chronic hyperglycaemia results in damage to various organs, particularly the eyes, kidneys, nerves, heart, and blood vessels^1^,^2^. Diabetes influences corneal structure and function, thereby causing epithelial defects, recurrent corneal erosion, delayed wound-healing, delayed re-epithelialisation, increased epithelial fragility, and altered epithelial and endothelial barrier functions, as well as other serious lesions^3^,^4^. Therefore, it is important to clarify and analyse the cellular and molecular mechanisms affecting the physiological status of the cornea in patients with diabetes.

The circadian rhythm is an endogenous biological rhythm that is associated with the 24-h day/night cycle. The energy balance and physiological activities of an organism, including feeding behaviour, glucose and lipid metabolism, hormone and neurotransmitter secretion, and sleep, are subject to the circadian rhythm^5^,^6^,^7^,^8^. The mammalian circadian clock system consists of a master clock in the hypothalamic suprachiasmatic nuclei as well as multiple peripheral clocks^8^. At the molecular level, circadian rhythms are controlled by the transcriptional/translational feedback loops of the circadian clock system. This system includes a set of core clock genes, such as *Clock*, *Bmal1*, *CRY* family genes, *PER* family genes, and nuclear receptors (*Rev-erbα* and *ROR*), that adapt to the external environment^9^. The circadian clock regulates metabolism by controlling key rate-limiting enzymes, transport systems, and nuclear receptors that are coupled to lipid and carbohydrate metabolism^8^,^10^,^11^.

Metabolic alterations can affect the circadian clock. For example, obesity alters *Clock* expression in the brainstem. In mice with high-fat diet-induced obesity, *Clock* expression levels are upregulated, *Cry1* expression is downregulated, and *Bmal1* and *Rev-erbα* expression levels are attenuated^12^. Streptozotocin (STZ)-induced diabetes promotes the advancement of phases of the circadian clock in the heart, resulting in the loss of normal synchronisation with the peripheral environment^13^.

The physiological functions of almost every system in the body are governed by circadian rhythms. The cornea is an important component of the dioptric media of the eye, contributing a third of the refractive power of the entire visual system. The normal growth and development of the cornea are subject to the regular diurnal rhythm of light and dark periods. In young chicks, normal ocular development is inhibited by exposure to constant light, resulting in severe corneal flattening and corneal thickening^14^. Corneal thickness exhibits regular diurnal variations; it is thickest at 6:00 AM and thinnest at 10:30 PM^15^. Corneal mitosis and DNA synthesis also have regular diurnal variations, with high activity from 5:00 AM to 9:00 AM^16^,^17^, and significant changes in the limbal epithelium have been noted^18^. The presence of a circadian clock in the cornea has been shown in luciferase-reporter transgenic mouse lines^19^. Nonetheless, the effects of diabetes on the physiological circadian rhythm in the cornea remain unknown.

Meanwhile, the mammalian immune system^20^ and many immune components^21^,^22^,^23^,^24^,^25^, including macrophages, natural killer cells, spleen, and lymph nodes, also exhibit diurnal variations. Neutrophils play an important role in maintaining the immune functions of the body by conferring resistance to microbial infection. The migration of neutrophils to normal peripheral tissue exhibits rhythm under physiological conditions^26^ and is controlled by adrenergic nerves. In addition, the migration of neutrophils towards an injured area exhibits robust rhythmicity that peaks in darkness^27^. Moreover, neutrophils in diabetic rats show abnormal leukostasis, such as increased diabetic retinal leukostasis^28^. Gamma-delta T-cells, which are the main lymphocytes of innate immunity, are present in the normal conjunctiva and limbal regions, including the ocular surface epithelium. These cells can stimulate the division of the corneal epithelial cells through the secretion of interleukin (IL)-22^29^. In addition, γδ T-cells can produce IL-17 as a chemotactic signal to attract peripheral blood myeloid cells such as neutrophils and monocytes^30^. After corneal abrasion, γδ T-cells are necessary for normal corneal wound-healing because they stimulate platelet and neutrophil accumulation in the wounded cornea^30^.

There is a close bidirectional relationship between metabolism and circadian rhythms^31^. Circadian-rhythm disturbances interfere with normal metabolism and promote the development of certain metabolic diseases, such as obesity and diabetes^32^. In contrast, alterations in the metabolic state, such as a high-fat diet^33^ and changes in feeding times^34^, affect the normal circadian clock rhythm. *Bmal1* expression is downregulated in the livers of rats with STZ-induced type 1 diabetes^35^. In addition, an increased glucose concentration downregulates *Per2* expression in cultured Rat-1 fibroblasts^36^. In diabetic mice, *Per2* mRNA levels are downregulated in the liver^37^,^38^ and kidney^39^, and the degree of downregulation is associated with disease severity^40^. However, the effect of insulin-dependent diabetes on the circadian profile of the cornea remains unknown.

Insulin has recently been shown to induce a phase shift in the peripheral clock by advancing the phase of the feeding-related clock^41^. Meanwhile, early insulin treatment could prevent the disruption of the body temperature rhythm in diabetic animals^42^. It is unclear whether the abovementioned rhythm alterations—those of circadian clock system genes, mitosis, and cell migration—can be improved or restored by insulin.

In the present study, mice were injected with STZ, which destroys insulin-producing β cells in the pancreas^43^, to induce diabetes. These mice were used to investigate the circadian rhythm of the cornea via the analysis of clock gene expression, mitosis, and leukocyte-trafficking to the limbal region. The potential effects of early insulin administration on the abovementioned corneal rhythms in diabetic mice were also examined.

## Results

**Figure 1:**
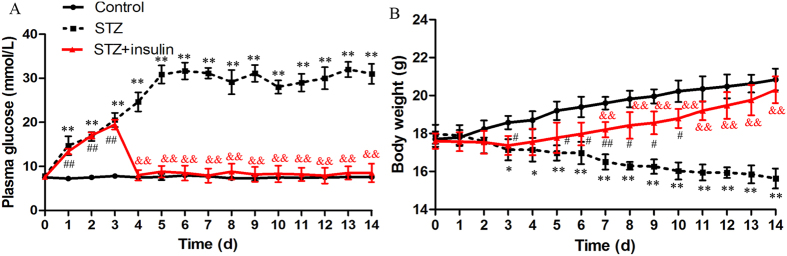
Effects of streptozotocin (STZ) on plasma glucose levels and body-weight development of C57/BL6 mice. Values are the mean ± standard deviation of control, STZ-treated, and STZ + insulin animals. Insulin was administered on the 3rd day after STZ injection. (**A**) Plasma glucose levels and (**B**) body weights in the control, STZ, and STZ + insulin groups are shown (n = 5 mice per group). Student’s t-tests were used to analyse differences between groups. *P < 0.05, **P < 0.01, STZ group vs. Control group; ^#^P < 0.05, ^##^P < 0.01, STZ + insulin group vs. Control group; ^&^P < 0.05, ^&&^P < 0.01, STZ group vs. STZ + insulin group.

Diabetes was induced by a single intraperitoneal injection of STZ. At 24 hours after the injection, blood glucose levels increased from 7.80 ± 0.28 mmol/L to 14.72 ± 2.13 mmol/L. These levels stabilised and remained high at 31.00 ± 2.30 mmol/L at 14 days (Fig. 1A). The blood glucose levels of control mice remained relatively constant throughout this period. The control mice maintained body weights of linear growth. In contrast, the body weights of STZ-treated mice decreased significantly compared with those of control mice after 3 days (Fig. 1B), declining to 15.63 ± 0.53 g at 14 days. This decline is consistent with the STZ-induced diabetes model^44^. After the administration of insulin to diabetic mice, blood glucose levels significantly decreased, and body weights increased to baseline values.

### Insulin can restore the altered rhythm of clock genes in epithelial cells in the diabetic cornea

**Figure 2:**
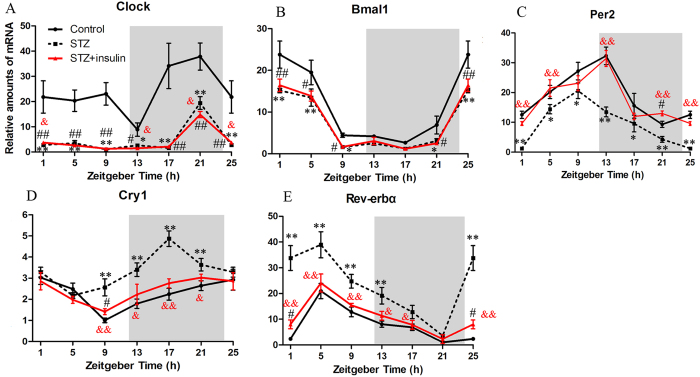
Effect of insulin on the circadian rhythm of clock gene expression in the diabetic cornea. Expression levels of *Clock* (**A**) *Bmal1* (**B**) *Per2* (**C**) *Cry1* (**D**) and *Rev-erbα* (**E**) in corneas isolated from control, STZ-treated, and STZ + insulin mice during the 24-h light/dark cycle. Values are the mean ± standard deviation and were normalised against the amount of glyceraldehyde 3-phosphate dehydrogenase mRNA, with minimum values defined as 1. One-way ANOVA shows significant rhythmicity of the five genes in the control group (n = 3 independent experiments, each experiment contain 6 corneas in each group). Factorial-design ANOVA was performed to analyse differences between two groups, and a Student’s t-test was used to compare replicates by time-point. The grey area denotes the dark phase. *P < 0.05, **P < 0.01 STZ group vs. Control group; ^#^P < 0.05, ^##^P < 0.01 STZ + insulin group vs. Control group; ^&^P < 0.05, ^&&^P < 0.01, STZ group vs. STZ + insulin group.

To test the hypothesis that changes in diabetes-associated metabolic states can affect the circadian rhythms of corneal physiology, we examined the cycling patterns of clock genes and mitosis in the corneal epithelium of diabetic animals, as well as the trafficking of neutrophils and γδ T-cells. Circadian clocks are endogenous oscillators that drive daily physiological rhythms. The cell-autonomous clock is governed by an interlocking network of transcriptional feedback loops. Hundreds of clock-controlled genes regulate tissue-specific functions. *In vitro* and *in vivo* experiments show that the cornea also displays robust oscillations of the expression of some circadian genes^45^,^46^,^47^. To further confirm the circadian rhythm via clock-gene expression, we examined the mRNA levels of five clock genes (*Clock*, *Bmal1*, *Per2*, *Cry1*, and *Rev-erbα*) at 4-h intervals during the 24-h light/dark cycle. *Clock* (Fig. 2A), *Bmal1* (Fig. 2B), *Per2* (Fig. 2C), *Cry1* (Fig. 2D), and *Rev-erbα* (Fig. 2E) mRNA exhibited well-defined, 24-h rhythmicity in the normal cornea (one-way ANOVA, P < 0.01). *Clock* had a peak at ZT21 and a trough at ZT13, *Bmal1* had a peak at ZT1 and a trough at ZT17, *Per2* had a peak at ZT13 and a trough at ZT21, *Cry1* had a peak at ZT25 and a trough at ZT9, and *Rev-erbα* had a peak at ZT5 and a trough at ZT21.

Next, we examined whether the above fluctuations in the expression of the five clock genes were different in STZ-induced diabetic mice. The expression levels of *Clock* (factorial design ANOVA, P < 0.01; Fig. 2A), *Bmal1* (factorial design ANOVA, P < 0.01; Fig. 2B), *Per2* (factorial design ANOVA, P < 0.01; Fig. 2C), *Cry1* (factorial design ANOVA, P < 0.05; Fig. 2D), and *Rev-erbα* (factorial design ANOVA, P < 0.01; Fig. 2E) significantly differed between control and STZ-induced diabetic mice. The expression levels of *Clock*, *Bmal1*, and *Per2* were significantly decreased in STZ-induced diabetic mice, but the levels of *Cry1* and *Rev-erbα* were significantly increased. These results show that insulin deficiency alters the expression of most clock genes.

Insulin was previously found to reset peripheral clocks in response to feeding cues^48^. In addition, insulin rapidly affects the expression of *Per1*, *Per2*, and *Dec1* in cultured rat hepatocytes^49^. In agreement with cell-culture studies, *in vivo* experiments have shown that an insulin injection elicits a rapid change in *Per2* and *Rev-erbα* expression and shifts the phase of the clocks of insulin-sensitive tissue (e.g., muscle and adipose tissues), but not those of insulin-insensitive tissue (e.g., lungs)^50^. Thus, the effect of insulin on peripheral clocks is tissue-specific. The present study confirmed that insulin restores the circadian rhythm in *Per2* (factorial design ANOVA, P < 0.05; Fig. 2C), *Cry1* (factorial design ANOVA, P < 0.05; Fig. 2D), and *Rev-erbα* (factorial design ANOVA, P < 0.05; Fig. 2E), and the difference between the normal control group and insulin treatment group was not significant. However, insulin did not restore the circadian rhythm in *Clock* (factorial design ANOVA, P > 0.05; Fig. 2A) and *Bmal1* (factorial design ANOVA, P > 0.05; Fig. 2B), with the analysis showing statistically significant differences between the insulin-treated diabetes and control groups. These results show that the cornea was insulin-sensitive. Furthermore, insulin acted to reset the circadian oscillation in the diabetic cornea.

### Alteration in division rhythm of diabetic corneal epithelial cells

**Figure 3:**
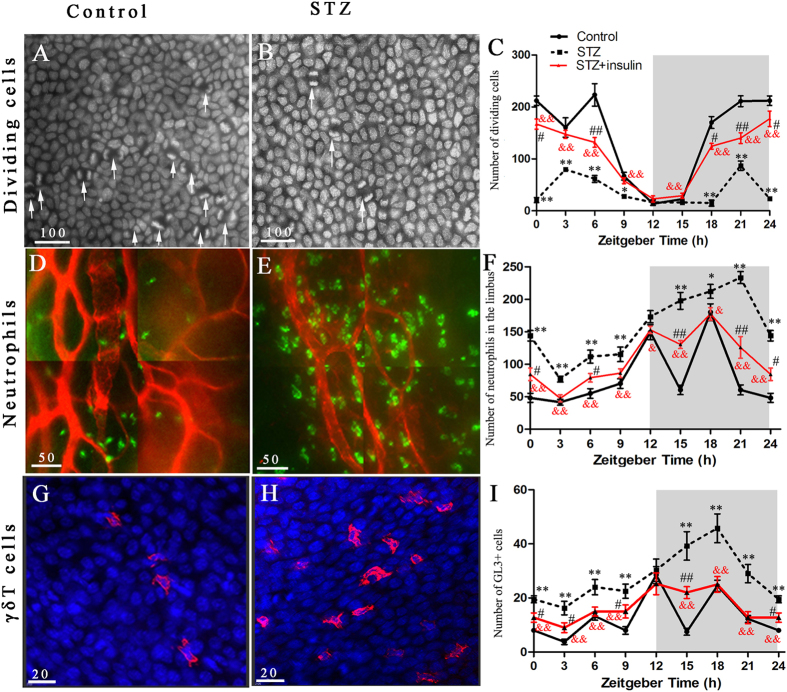
Effect of insulin on circadian rhythms in the mitotic division of the diabetic corneal epithelium and the recruitment of neutrophils and γδ T-cells to the diabetic corneal limbus. (**A**,**B**) Photographs of the dividing cells in the corneal epithelium at Zeitgeber Time (ZT) 6 from control corneas and diabetic corneas. White arrows indicate the dividing cell with paired nuclei (DAPI staining; original magnification: 400x). (**C**) Time course of dividing epithelial cells in control, STZ, and STZ + insulin groups. (**D**,**E**) Photomicrographs of the limbus showing control corneas and diabetic corneas at ZT 15 stained with FITC-labelled anti-Ly6g (to identify neutrophils) and PE-labelled anti-CD31 (to identify vessel endothelium). (**F**) Time course of neutrophil recruitment to limbus from blood in control, STZ, and STZ + insulin groups. (**G**,**H**) Photomicrographs of the limbal epithelium showing control corneas and diabetic corneas at ZT 15 stained with anti–Gl3^+^-PE (to identify γδ T-cells) and DAPI (to identify nuclei). (**I**) Time course of γδ T-cell recruitment to limbal epithelium in control, STZ, and STZ + insulin groups (n = 5 corneas per group). The grey area denotes the dark phase. *P < 0.05, **P < 0.01, STZ group vs. Control group; ^#^P < 0.05, ^##^P < 0.01, STZ + insulin group vs. Control group; ^&^P < 0.05, ^&&^P < 0.01, STZ group vs. STZ + insulin group.

To investigate whether dividing cells in the corneal epithelium show circadian rhythm, we analysed mitotic cell-counts in the corneal epithelium at 3-h intervals during the 24-h light/dark cycle. Dividing cells were detected using DAPI staining (Fig. 3A,B). The circadian profiles of the dividing cells in normal control mice showed diurnal changes (one-way ANOVA, P < 0.01; Fig. 3C). Dividing cells peaked from ZT18 to ZT6 and showed a trough from ZT9 to ZT15.

The circadian profiles of the dividing cells were altered in diabetic mice compared with those in control animals (factorial design ANOVA, P < 0.01; Fig. 3C). Control mice had peak levels of dividing cells at the transition from dark to light (ZT0), whereas diabetic mice had peak levels at ZT3 and ZT21. In addition, the numbers of dividing cells in diabetic mice were significantly decreased at ZT0 (P < 0.01), ZT3 (P < 0.01), ZT6 (P < 0.01), ZT9 (P < 0.05), ZT18 (P < 0.01), and ZT21 (P < 0.01).

Treatment with insulin significantly restored the rhythm amplitude of dividing cells in diabetic mice, although normal control levels were not reached (factorial design ANOVA, P < 0.05; Fig. 3C).

### Insulin restores the altered rhythmic recruitment of leukocytes to the corneal limbal region in diabetic corneas

Limbal neutrophils were abundantly stained with Ly6g (Fig. 3D,E). The circadian profiles of limbal neutrophils in control mice showed diurnal changes (one-way ANOVA, P < 0.01; Fig. 3F). γδ T-cells were revealed by GL3 staining (Fig. 3G,H). The circadian profiles of γδ T-cells in control mice also showed diurnal changes (one-way ANOVA, P < 0.01; Fig. 3I).

The circadian profiles of limbal neutrophils were significantly altered in diabetic mice (factorial design ANOVA, P < 0.05; Fig. 3F). Control mice exhibited peak neutrophil levels at ZT12 and ZT18, whereas diabetic mice exhibited peaks at ZT21. The number of neutrophils in diabetic mice was significantly increased. The circadian rhythm of limbal γδ T-cells was altered in diabetic mice compared with that in control mice (factorial design ANOVA, P < 0.01; Fig. 3I). Control mice exhibited two γδ T-cell peaks at ZT12 and ZT18, whereas diabetic mice exhibited a single γδ T-cell peak at ZT18. Compared with the controls, the number of γδ T-cells in diabetic mice was significantly increased at ZT0, ZT3, ZT6, ZT9, ZT15, ZT18, and ZT21 (all P < 0.01). These data suggest that the diabetic environment facilitates circadian-based leukocyte-trafficking to the cornea.

Furthermore, treatment of diabetic mice with insulin affected the circadian rhythm of limbal neutrophils (factorial design ANOVA, P < 0.01; Fig. 3F) and shifted peak neutrophil levels from ZT21 to ZT18. However, it did not reach the level observed in control mice (factorial design ANOVA, P < 0.01) and was roughly situated in the middle of the control and diabetic groups. Treatment of diabetic mice with insulin also affected the circadian rhythm of γδ T-cells (factorial design ANOVA, P < 0.01; Fig. 3I), although their levels did not reach those of control mice (factorial design ANOVA, P < 0.01). These results indicate that early insulin treatment reversed the abnormal inflammation-like state.

## Discussion

Diabetes affects corneal morphology, metabolism, and physiology. It can also cause diabetic keratopathy^51^. More than 70% of diabetic patients develop diabetic keratopathy and show some morphological changes in their cornea^52^. Documented diabetic keratopathy changes include epithelial defects, recurrent epithelial erosions, delayed wound-healing, and ulcers. However, the cellular and molecular mechanisms underlying these events are poorly understood. In this study, we show that diabetes influenced the circadian rhythm in the expression of five core clock genes (*Clock*, *Bmal1*, *Per2*, *Cry1*, and *Rev-erbα*), as well as mitosis, in the normal murine corneal epithelium. Diabetes also increased the emigration fluctuation of neutrophils and γδ T-cells to the limbal region. Furthermore, the early administration of insulin to diabetic animals partially restored the alterations in rhythmic activity in the murine cornea.

Core clock genes are the essential elements of circadian rhythm. Through positive and negative feedback loops, the circadian clock regulates the physiological activities of almost all cells, tissues, and organs. Similar to other systems, we show that the expression of five clock genes (*Clock*, *Bmal1*, *Per2*, *Cry1*, and *Rev-erbα*) in the normal mouse cornea had a robust circadian fluctuation, which was consistent with several recent observations^45^,^46^,^47^. Diabetes altered the circadian rhythm of the corneal epithelium in mice, such that the expression of *Clock, Bmal1*, and *Per2* was downregulated, and the expression of *Cry1* and *Rev-erbα* was upregulated. However, the significance of the altered expression levels of these genes and the mechanisms associated with corneal pathological changes remain unclear.

The present study also demonstrates that insulin treatment restores the expression rhythm of *Per2*, *Cry1*, and *Rev-erbα*. These findings are consistent with the ability of insulin to induce a phase shift in the peripheral clock, thereby advancing the phase of the feeding-related clock^41^ to correct the impaired rhythm of Per2 expression in the liver^41^ and to partially normalise the heat rhythm in rats with STZ-induced diabetes^53^. Our findings also suggest that insulin is able to synchronise the altered corneal circadian clock in diabetic animals. However, our data indicate that insulin supplementation by intraperitoneal injection has no effect on the circadian expression of *Clock* and *Bmal1*. A possible explanation for this finding might be that insufficient time had elapsed for the insulin treatment to allow the complete restoration of the expression of these genes.

The thickness of the normal corneal epithelium is largely maintained by continuous mitosis and basal-cell migration to the surface. Traditionally, it is hypothesised to be dependent on three factors, termed the XYZ hypothesis by Thoft and Friend^54^, where X represents the proliferation and anterior migration of basal epithelial cells, Y represents basal-cell migration from the periphery to the centre of the cornea, and Z represents the desquamation of epithelial cells from the surface. Based on this hypothesis, corneal epithelial mitosis is a core factor that maintains corneal epithelial integrity and homeostasis. In the present study, we showed diurnal changes in normal mouse corneal mitosis, which was in accordance with previous studies showing that corneal mitosis and DNA synthesis exhibit circadian oscillations associated with the changes in light during the day and night^16^,^17^,^18^. However, the diabetic condition decreased the circadian rhythm of corneal epithelial mitosis. This decline in the ability of cells to divide may provide a new clue for why diabetic patients are prone to corneal epithelial lesions. In addition, circadian rhythms influence generative processes after wounding^55^. Corneal wounding at different times of the day reveals that the regenerative response of the corneal epithelium to injury is time-dependent^18^. Moreover, epithelial proliferation is significantly altered^56^. Hence, our results provide another explanation for delayed corneal wound-healing after the development of diabetes. Insulin treatment was able to partially normalise the circadian rhythm of mitosis. Therefore, we hypothesise that, given the diurnal changes in corneal epithelial cell mitosis in animals with insulin-dependent diabetes, the altered self-renewal speed could contribute to delayed corneal wound-healing in diabetes. Earlier studies have shown that insulin treatment ameliorates impaired corneal re-epithelialisation in diabetic rats^57^, and that topical application of insulin in rats with type 1 diabetes mellitus normalises corneal wound-healing^58^.

Furthermore, leukocyte migration to peripheral tissues under homeostasis is critical for surveilling and combating pathogens. Recent evidence has shown that the immune system is controlled by the circadian clock, with oscillations in the number and activity of immune cells in the blood and tissues over the course of 24 hours^20^,^59^. Consistent with this observation, we found diurnal rhythms in immune cell-trafficking to the corneal limbal region. However, diabetes altered the circadian rhythm of neutrophils’ migration to the limbus. The peak of neutrophil trafficking time was shifted in diabetic mice. More importantly, the peak was significantly higher than that in the normal control group. This finding is consistent with studies of diabetic retinopathy, which have shown an increased number of static leukocytes in STZ-induced diabetic rats and neutrophil priming^60^. The latter results in higher levels of superoxide^61^,^62^ and cytokines^63^. Neutrophils are the main immune cells in the early phase of corneal wound-repair^64^ and are essential for controlling and eliminating microbial infections from wound areas via phagocytosis, degranulation, and the release of neutrophil elastase and reactive oxygen species. However, there are two sides to neutrophils. For example, aberrant neutrophil-migration to the wound delays corneal wound-healing^65^. Thus, dysfunctional neutrophil-trafficking to the cornea induces an inflammatory state. Our data show that insulin administration at an early stage could reverse this abnormal inflammatory state.

γδ T-cells are a lineage of innate-like lymphocytes that connect the innate and adaptive immune inflammatory responses^66^. On the ocular surface, γδ T-cells constitutively express IL-17^30^, which acts as a chemotactic factor to attract peripheral blood myeloid cells, such as neutrophils^67^ and monocytes^68^. We found that γδ T-cell recruitment to the corneal limbal region, including both epithelial and stromal layers, showed a circadian rhythm that was shifted and increased by diabetes. The increase in γδ T-cell numbers may explain the inflammatory state in the diabetic cornea. In addition, systemic insulin-administration partially normalised the level and pattern of leukocyte-trafficking to the cornea. Therefore, the lack of insulin brought more immune cells to the cornea by altering the circadian systems. This inflammatory state may potentially contribute to the delay in corneal wound-healing in diabetes.

In summary, we show that insulin-dependent diabetes alters the corneal circadian rhythm, thus affecting the expression of the main core clock genes, the mitosis of corneal cells, and the trafficking of leukocytes to the cornea. Insulin administration partially normalises the rhythmic pattern and amplitude and reverses the deviant inflammatory state in unwounded eyes. These observations showed new light on the poorly understood pathophysiologic mechanisms of diabetic keratopathy. However, the mechanism responsible for the efficacy of therapeutically administered insulin still needs to be determined. Overall, this study might have important implications for the identification of new targets for improving diabetic keratopathy.

## Methods

### Diabetes model and insulin treatment

All of the animal protocols were approved by the Jinan University Laboratory Animal Committee for Animal Welfare. All animals were treated in accordance with the Association for Research in Vision and Ophthalmology Statement for the Use of Animals in Ophthalmology and Vision Research and the guidelines of the Animal Experimental Committee at Jinan University. Animals were anesthetised using short inhalational anaesthesia with 2% isoflurane for minor procedures. Animals were euthanised via CO2 overdose and cervical dislocation. Female C57/BL6 mice (7 weeks old) with no eye diseases were purchased from the Animal Experimental Centre of Guangdong Province. Mice with body weights of 17.70 ± 0.40 g and blood glucose levels of 7.83 ± 0.29 mmol/L were used for diabetes induction. The mice were randomly divided into three groups: control, STZ, and STZ + insulin groups. Prior to STZ injection, no significant differences in blood glucose levels (P = 0.469) or body weight (P = 0.591) were noted amongst the three groups.

STZ was dissolved in citrate buffer. Diabetes was induced by a single intraperitoneal injection of 150 mg/kg STZ (Tocris, USA)^38^. On the third day after STZ injection, some of the STZ-treated rats received 20 mg/kg insulin (Sigma, USA) via subcutaneous injections at sunrise and sunset; this treatment pattern continued for the following 11 days^38^. Control animals were injected with an equivalent volume of citrate buffer. All animals were maintained on a 12-hour light/dark cycle. The lights went on at 7:00 AM (Zeitgeber Time [ZT] 0) in a temperature-controlled room (23 ± 2 °C), and the animals were provided food and water *ad libitum*. Blood glucose concentrations were analysed with an Accu-Chek Active glucometer (Roche, Germany) glucose analyser once a day after STZ injection. Hyperglycaemia was defined as blood glucose concentrations ≥11.10 mmol/L^69^. All corneas were acquired on Day 14.

### RNA isolation and quantitative real-time polymerase chain reaction (PCR)

The control, STZ, and STZ + insulin groups were euthanised via cervical dislocation every 4 hours throughout the 24-h light/dark cycle; this began at 1 h (ZT1) after the lights went on. The eyeballs were trimmed under a stereoscopic microscope (OLYMPUS SZ61, Japan) to obtain the corneal tissue with the limbus. Corneal epithelial cells were obtained by mechanical scraping with a golf-club spud scraper (Accutome, USA) after dropping 10 ul TRIzol reagent (RNAsimple Total RNA Kit, TIANGEN, Japan) on the corneal surface, and were homogenised in TRIzol reagent (RNAsimple Total RNA Kit, TIANGEN, Japan). RNA was isolated according to the manufacturer’s instructions. Total RNA was reverse-transcribed using random primers and reverse transcriptase, according to the manufacturer’s protocol (ReverTra Ace qPCR RT Kit, TOYOBO, Japan). Complementary DNA (cDNA) was mixed with SYBR Green PCR Master Mix (THUNDERBIRD SYBR qPCR Mix, TOYOBO, Japan) and different sets of gene-specific forward and reverse primers (Table 1). The reactions were performed using a real-time PCR system (Bio-Rad, USA), and all reactions were performed in triplicate. Data were normalised to the level of glyceraldehyde-3-phosphate dehydrogenase (GAPDH).

### Whole-mount technique and immunofluorescence staining

The experiments were performed according to a standard protocol^70^. Various antibodies were used for immunofluorescence staining. The entire eyeball was removed under a stereo microscope and fixed in 2% paraformaldehyde in phosphate-buffered saline (PBS) for 40 minutes. The corneas were trimmed from the eyeball, washed three times in PBS for 5 minutes each time, blocked in 0.1 M PBS containing 2% bovine serum albumin (BSA) for 15 minutes, and permeabilised with 1% Triton X-100 in BSA/PBS for 15 minutes. Afterwards, the corneas were incubated in 0.1 M BSA/PBS and 0.1% Triton X-100 with anti-Ly6g fluorescein isothiocyanate (FITC, 3:100; BD Biosciences, USA) or anti-CD31-PE (3:100, BD Biosciences, USA) or anti–TCRδ-PE (clone GL3, 3:100, BD PharMingen, USA) for 24 h at 4 °C to detect neutrophils, vessel endothelium, or γδ T-cells, respectively. After incubation, the tissues were washed in 0.1 M PBS three times for 5 minutes each time. The whole cornea was cut into four quadrants, then stretched and mounted on a slide using anti-fade mounting media with 1 μM 4,6-diamidino-2-phenylindole (DAPI) (Sigma-Aldrich, USA) overnight, and then stored in the dark.

### Counting of dividing corneal epithelial cells and γδ T-cells

**Figure 4:**
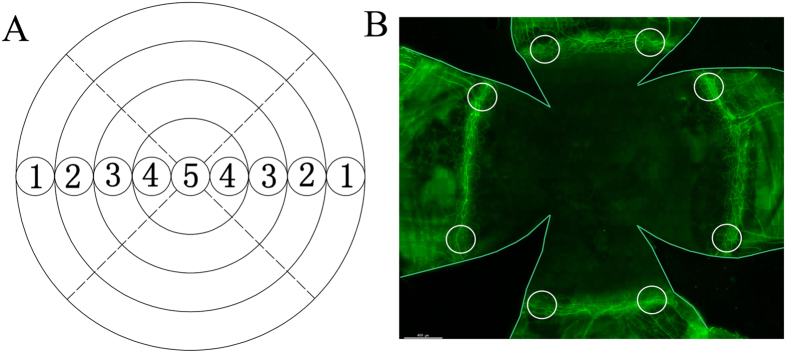
Microscopic fields examined during the analysis of the cornea. (**A**) Morphometric analysis showing patterns of dividing cells across the cornea in nine 40× fields. (**B**) Circles depicting eight regions sampled to quantify neutrophils and γδ T-cells in 40× fields.

Whole corneas with the complete limbus from different circadian time-points were mounted with the epithelium facing upwards on a glass slide^71^. Each experimental group included at least four to six corneas. To evaluate mitosis in the corneal epithelium, two cornea diameters were selected, and the number of dividing cells with DAPI-stained and paired nuclei in the corneal basal cell layer were counted from one side of the limbus to the other using a DeltaVision Image System^72^ (Fig. 4A). These paired cells comprised 97.8% to 98.3% of BrdU-labelled cells^73^. Neutrophils around the limbal vessels were counted in eight different regions of the corneal limbus under 40X microscope (Fig. 4B). γδ T-cells in the epithelial layer and the stromal layer were counted in eight different regions of the corneal limbus (Fig. 4B).

### Statistical analysis

Data are presented as the means ± standard deviation. Data were analysed using the SPSS 21.0 software package. Body weights and blood glucose concentrations were analysed using Student’s t-tests. The 24-h rhythms were analysed using one-way ANOVA. The statistical analysis was performed using factorial design ANOVA to compare the data obtained from independent samples. P < 0.05 was considered significant.

## Additional Information

**How to cite this article**: Song, F. *et al*. Insulin Restores an Altered Corneal Epithelium Circadian Rhythm in Mice with Streptozotocin-induced Type 1 Diabetes. *Sci. Rep*. **6**, 32871; doi: 10.1038/srep32871 (2016).

## Figures and Tables

**Table 1:** Real-time RT-PCR primers.

Gene	Primer Sequence	Temperature	Length (bp)
*Clock*	5′-CGCTCCCGTGAAAGAAAA-3′	55 °C	300
	5′-CTTCTTCCACCAATCCATCA-3′		
*Bmal1*	5′-AGAGCTTGTTTGACTACCTG-3′	55 °C	247
	5′-TTCTTTGAACAGGTAGAGGC-3′		
*Per2*	5′-CGCCTAGAATCCCTCCTGAGA-3′	60 °C	202
	5′-CCACCGGCCTGTAGGATCT-3′		
*Cry1*	5′-CAGACTCACTCACTCAAGCAAGG-3′	60 °C	126
	5′-TCAGTTACTGCTCTGCCGCTGGAC-3′		
*Rev-erbα*	5′-AAGGTTGTCCCACATACTTC-3′	55 °C	383
	5′-GTTTTTCAGACACCGTTTGT-3′		
*GAPDH*	5′-CAAGGACACTGAGCAAGAG-3′	57 °C	151
	5′-TGCAGCGAACTTTATTGATG-3′		

GAPDH: glyceraldehyde 3-phosphate dehydrogenase.
